# Comparative Study of the Morphology of Cellulose Nanofiber Fabricated Using Two Kinds of Grinding Method

**DOI:** 10.3390/ma15207048

**Published:** 2022-10-11

**Authors:** Khulan Uranchimeg, Battsetseg Jargalsaikhan, Amgalan Bor, Kiyoung Yoon, Heekyu Choi

**Affiliations:** 1 Graduate School of Material Science Engineering, Changwon National University, Changwon 641-773, Gyoungnam, Korea; 2 Department of Chemical and Biological Engineering, School of Engineering and Applied Sciences, National University of Mongolia, Ulaanbaatar 14200, Mongolia; 3 R&D Center, REACNF Co., Ltd., Changwon 641-773, Gyoungnam, Korea; 4 Department of Mechatronics Convergence Engineering, College of Engineering, Changwon National University, Changwon 641-773, Gyoungnam, Korea; 5 Graduate School of Convergence on Culture Technology, Changwon National University, Changwon 641-773, Gyoungnam, Korea; 6 Research Institute of Future Convergence, Changwon National University, Changwon 641-773, Gyoungnam, Korea

**Keywords:** cellulose nanofiber (CNF), dry cellulose powder, planetary ball mill (PBM), disc grinder

## Abstract

In this paper, a comparison of cellulose nanofiber (CNF) fabrication from *Gelidium amansii* using two kinds of grinding processes is presented. The cellulose from *Gelidium amansii* is pretreated with hydrogen peroxide and sodium carbonate in a separating and bleaching process. Then, two grinding processes (method A and B) are used to fabricate CNFs. The first is a traditional method of fabricating CNFs using a disc grinder, whereas the second method is identical to the first, but includes an additional step involving a planetary ball mill. In the new method (method B), dry cellulose powder is prepared using a planetary ball mill, which has the advantage of long-term storage and maintains the original quality of the cellulose. The morphological changes of the dry cellulose powder and CNFs are determined using scanning electron microscopy and field emission scanning electron microscopy. The physical characteristics of the CNFs are found to be significantly different when we change the disc grinder used in the grinding method to produce nanometer scale where the best result is homogeneous, uniform CNFs with a fabricated width of 19 nm.

## 1. Introduction

Cellulose, composed of linear polymer chains of (C_6_H_10_O_5_)_n,_ is the most abundant organic polymer on Earth and can be extracted from a variety of sources, such as wood, bast fibers, grasses, seed fibers, marine animals, algae, fungi, invertebrates, and bacteria [1,2]. Cellulose material has attracted considerable attention due to its eco-friendly nature along with interesting physicochemical, biological, and mechanical properties in the form of nanocellulose [3]. The term “nanocellulose” is commonly used to characterize cellulose-based nanomaterials with at least one dimension in the nanometer range, and it mimics the tiniest structural component of diverse organisms’ cellulosic biomass [4].

Based on their cellulose properties [5], nanocelluloses can be divided into three categories: cellulose nanofibers (CNFs), cellulose nanocrystals (CNCs), and bacterial nanocellulose (BNC). Although all types have different morphologies, particle sizes, polymerizations, and other properties due to differences in their sources and fabrication methods [6], they all have similar chemical compositions [7].

CNFs are cellulose fibers modified to nanometer sizes, which can be induced to have much higher mechanical properties in polymer matrices in comparison to common cellulose fibers because of their higher crystallinity and mechanical properties combined with low density, active interfaces, and high surface areas [8]. CNFs show both amorphous and crystallinity parts and have a larger surface area with a weblike structure compared to CNCs, making them an attractive reinforcing material for polymers and composites [9]. CNFs can also be fabricated using a combination of chemical and mechanical techniques, according to some researchers [10].

Mechanical processes constitute one approach to reducing cellulose fibers into CNFs, by which the isolation of cellulose fibrils is achieved by applying a high shear force to separate the cellulose fibers along the longitudinal axis [11]. The most commonly used mechanical processes include high-pressure homogenization, ultrasonication, grinding, and ball milling methods [11]. Wang et al. [12] studied the production of CNFs from bleached eucalyptus pulp using a commercial stone grinder. As a result of the grinding, two main structures were found: untwisted fibrils, and twisted and entangled nanofibers. Iwamoto et al. [1] studied the extraction of dissolved pulp after 1–30 passes at 1500 rpm by a disc grinder. Here, after five passes through the disc grinder, nanofibers with widths of 20–50 nm were fabricated, and further passes did not change the size of the nanofibers. There were no significant changes in the morphology and widths of the CNFs when the number of passes was increased [1]. Therefore, we selected one to five passes for further study.

Ball milling is another mechanical method that can defibrillate cellulose fibers, and various types of ball millers are widely used in industry and laboratories, such as planetary ball mills (PBMs), mixer ball mills, and vibration ball mills [11]. Zhang et al. [13] extracted CNFs with high dimensional homogeneity and average fiber diameters less than 100 nm via ball milling. Phanthong et al. [11] studied two general kinds of cellulose (cellulose powder and paper), which were selected as the raw materials for nanocellulose extraction. The raw materials were initially processed in a PBM, followed by wet ball-milled cellulose with a decreased acid concentration. Although the fabrication of nanocellulose with the assistance of ball milling started in the early 2010s [11], nanocellulose is still being fabricated under wet conditions. 

The advantages of mechanical methods include ease of operation, environmental friendliness, cost-effectiveness, reliability, and increase in surface area [14]. On the other hand, the process of fabricating nanocellulose by the mechanical method is usually carried out under wet conditions. This causes problems such as cellulose quality change, high amount of storage space, long-term storage of cellulose, and preparation of nanocellulose with different concentrations in the fabrication process. Therefore, a new method (method B) was investigated in this study to convert cellulose to dry cellulose powder after the pretreatment process and to simplify the fabrication process of CNF. The preparation of dry cellulose powder using a PBM has the advantages of long-term storage, retention of the original qualities of cellulose, and the formation of good suspension. CNFs have a wide range of applications in various fields of industry, such as pharmaceuticals, cosmetics, food, civil engineering, and biomedical materials [15,16,17]. CNF fabrication from pretreated dry cellulose powder in this new method has many advantages in the pharmaceutical, food, and cosmetic industries, such as producing CNF at a suitable concentration for their products and the use of uniform homogenous CNF to improve the quality of the product.

In this current work, a comparative study of two kinds of grinding methods is performed to fabricate homogeneous, uniform, and minimum nanoscale CNFs from Gelidium amansii for a wide range of applications. Two kinds of grinding methods are employed: a traditional method (method A) and the new method (method B), where the new method includes the additional preparation of dry cellulose powder using a planetary ball mill (PBM). Indeed, the fabrication of CNFs via a disc grinding process using dry cellulose powder under optimal experimental conditions with a PBM is a new method with its own originality. The physical characteristics of CNFs manufactured by the traditional and new methods are compared, and the mechanisms of the two grinding methods are described. 

## 2. Materials and Methods

The brown algae Gelidium amansii used in this work was collected from Jeju Island in Korea and was purchased from Milyang agar.

### 2.1. Preparation of Cellulose by Pretreatment Process

**Figure 1:**
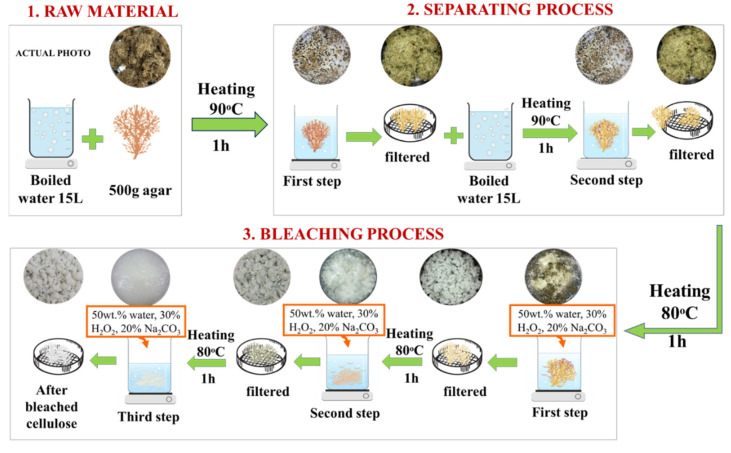
Scheme of the pretreatment process.

This section explains the pretreatment process shown in Figure 1. The Gelidium amansii was washed with water to remove all water-soluble compounds from the material. The sample was heated for one hour on a hot plate at 90 °C, and this process was repeated twice more. The Gelidium amansii was then submitted to an oxidative delignification process using hydrogen peroxide. This bleaching process was carried out in three steps, using 30% hydrogen peroxide (Sigma-Aldrich Co., Ltd., Burlington, MA, USA) and 20% sodium carbonate (Sigma-Aldrich Co., Ltd., Burlington, MA, USA) for 1 h at a temperature of 80 °C. Then, the pulp was filtered and washed with distilled water. During the last bleaching step, the pulp was washed with excess distilled water to remove the residual alkali. A pulp disintegrator (DAEIL Co., Ltd., Whasung, Korea) was used to disperse the cellulose sample in water at a cellulose content of 1 wt.%, and it was pulped 3000 times at 3000 rpm. 

### 2.2. Fabrication Method of CNF Using a Disc Grinder without a PBM (Method A)

**Figure 2:**
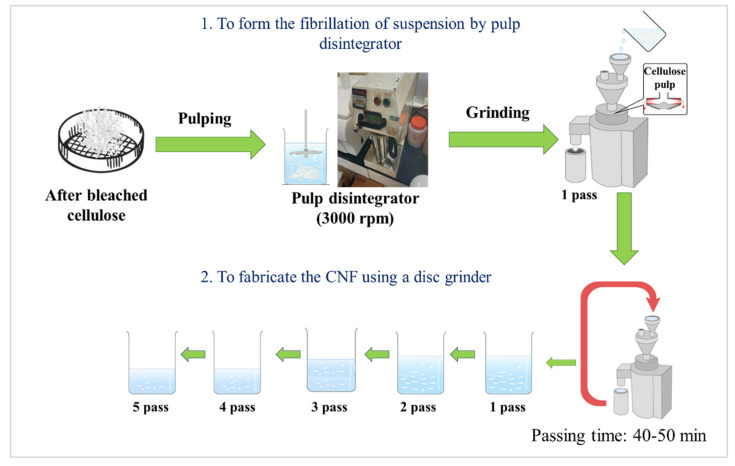
Scheme of method A.

The pretreated cellulose was processed according to Figure 2. The pretreated cellulose was fibrillated using suspension solids consisting of 1 wt.% with a disc grinder. This suspension was passed 1–5 times through a disc grinder at 1500 rpm for a repeated process. 

### 2.3. Fabrication Method of CNFs Using a Disc Grinder with a PBM (Method B)

**Figure 3:**
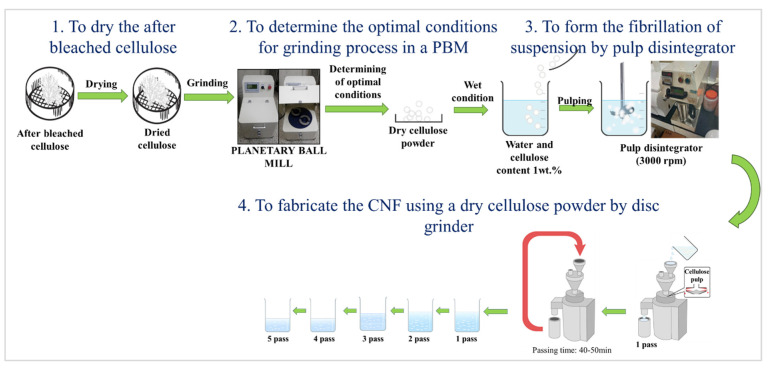
Scheme of method B.

After bleaching, the cellulose was dried and milled using a PBM. A suspension was formed using the prepared powder and then CNFs were extracted with a disc grinder, producing a dry cellulose powder. This continuous process is our new method, detailed in Figure 3. Preparation of the dry cellulose powder produced by different rotation speeds, ball powder ratios, ball diameters, and milling times were studied to determine the optimal conditions for the grinding process with the PBM. The optimal experimental conditions are shown in Table 1. 

Zirconia balls were used in this study because they are non-sensitive, have less chemical reaction, and have high density among ceramic balls. The dry cellulose powder was prepared in water with a cellulose sample proportion of 1 wt.%. The sample was disintegrated into a fibrous state using a pulp disintegrator at approximately 3000 rpm. A disc grinder was then used to make the CNFs from the dry cellulose powder with an extra cycle of 1–5 passes at 1500 rpm in the wet condition.

### 2.4. Morphology Characterization

Scanning electron microscopy (SEM) images of the obtained dry cellulose powders were taken to characterize their morphologies using a JSM-6510 (JEOL Ltd., Tokyo, Japan). All micrographs were taken at a magnification of between ×200 to ×1000 and an accelerating voltage of 5 kV. 

The fibrillated CNFs were also observed using a field emission scanning electron microscope, FE-SEM (JSM-7900F, JEOL Ltd.). The samples were prepared on a 32 mm diameter tube, coated with platinum sputter. The micrographs were collected ×100,000 magnifications.

## 3. Results and Discussion

### 3.1. Pretreatment Process

**Figure 4:**
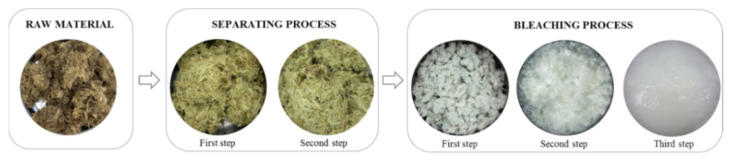
Photographs of the pretreated cellulose.

In the pretreatment process, the extraction procedures were common with three steps: a separating process to remove water-soluble compounds; a bleaching process to remove lignin by hydrogen peroxide; and alkali treatment to remove hemicellulose by sodium carbonate [18,19,20]. A principal goal in the bleaching process of cellulose is to remove lignin and hemicellulose without changing the cellulose’s properties [12]. Figure 4 shows the color change at each step of the pretreatment process. The lignin affects the high-quality properties of the raw material and decreases the pulp brightness level [21], which causes the color to change at each step of the pretreatment process.

**Figure 5:**
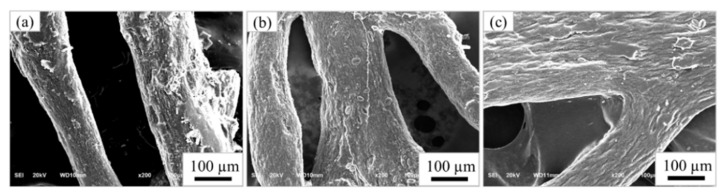
SEM micrographs (×200) of raw material (**a**) and pretreated sample, for the (**b**) first step and the (**c**) second step.

**Figure 6:**
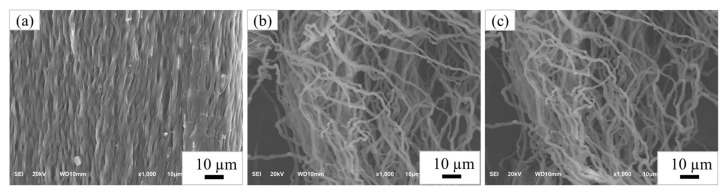
SEM micrographs (×1000) of bleached sample (**a**) first step, (**b**) second step, and (**c**) third step.

The morphology of the extracted cellulose after the bleaching process was observed using SEM. Figure 5 shows SEM images of the raw material and samples obtained from the separating process, which were taken at low magnification. As the SEM images show, the surface of the cellulose changed compared to the raw material due to the removal of water-soluble substances by the separating process [19]. Figure 6 shows SEM images of the samples after the bleaching process, which were taken at high magnification. At each step of the bleaching process, the morphology of the cellulose changed into a fiber structure because the hydrogen peroxide removed lignin from the cellulose and increased the whiteness of the cellulose [12,19,21]. 

From a morphological aspect, the alkaline and peroxide treatments were able to reduce the diameter size of the fibers, which indicates the removal of impurities (hemicellulose and lignin) from the fibers [20,22].

**Figure 7:**
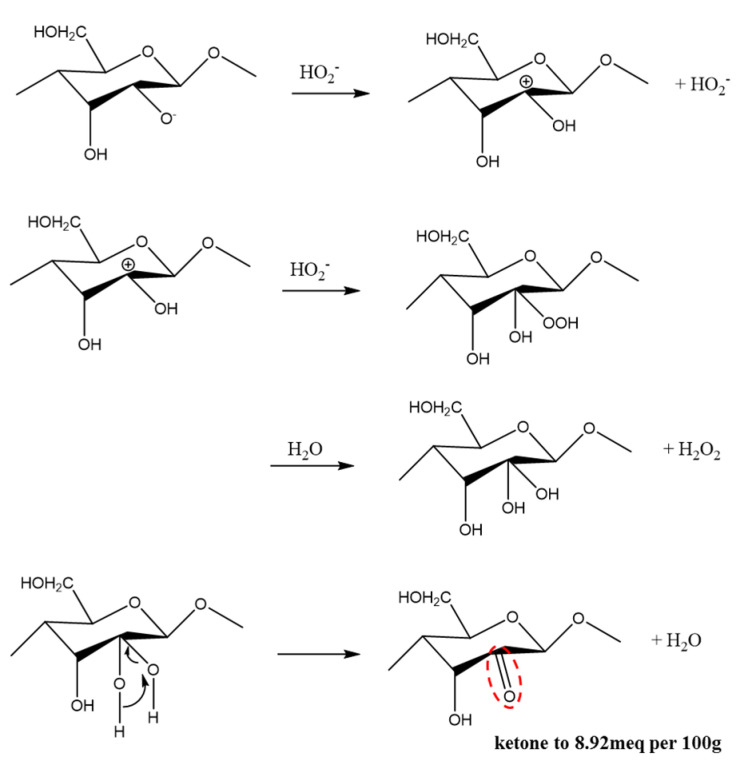
Oxidation of cellulose by hydroperoxyl radicals.

**Figure 8:**
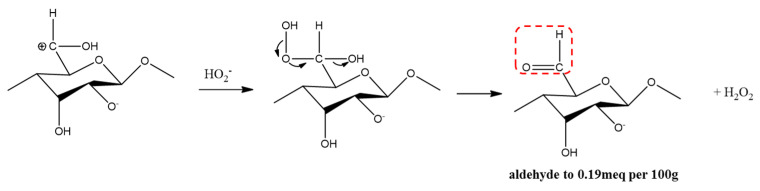
The oxidation to aldehyde.

**Figure 9:**
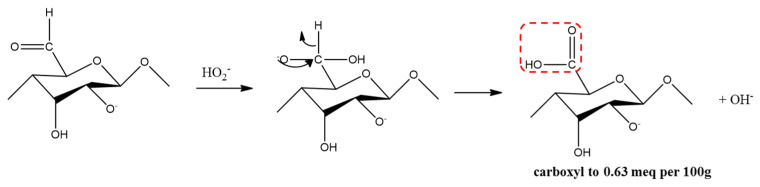
The oxidation to carboxylic acid.

Hydroperoxyl anions are strong nucleophiles and can react with α-β unsaturated ketones (Figure 7), including aldehyde (Figure 8) and carboxyl (Figure 9). Bleaching is often accomplished by oxidizing water-insoluble lignin to break chosen bonds and produce water-soluble compounds such as aromatic aldehydes and carboxylic acids [20].

Looking at the results of the pretreatment process, the color change of the raw material at each step of this process is due to the oxidation reaction (Figure 7, Figure 8 and Figure 9) effect of the bleaching process. As SEM images of the pretreatment process show, the morphology and size of the cellulose changed due to the bleaching process. The reason for this result is that the alkaline and peroxide treatments were able to reduce the diameter of cellulose and increase the whiteness of the cellulose.

### 3.2. FE-SEM Micrographs of the CNFs Produced by Just the Disc Grinder (Method A)

**Figure 10:**
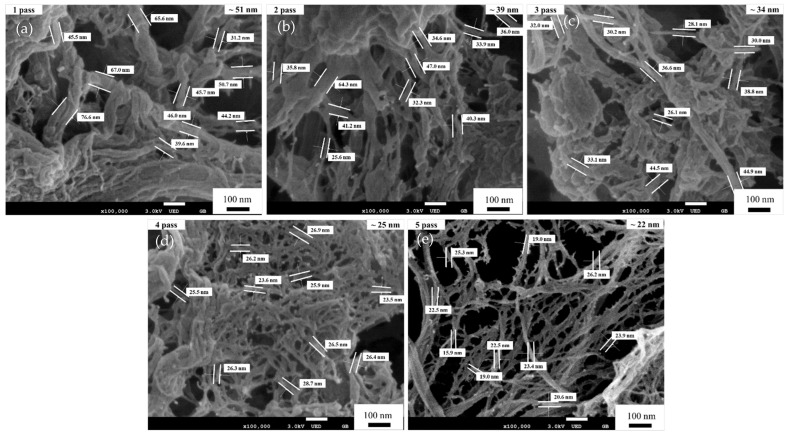
FE-SEM micrographs (×100,000) of CNFs after disc grinding without a PBM for (**a**) one pass, (**b**) two passes, (**c**) three passes, (**d**) four passes, and (**e**) five passes.

After the pretreatment process, the pulp fibers (cellulose suspension) were dispersed in water at a cellulose content of 1 wt.% using a pulp disintegrator. This cellulose suspension was passed through a disc grinder one to five times for repeated processing to fabricate the CNFs. Figure 10 shows FE-SEM images of the fibrillated pulp fibers after one to five passes through the disc grinder. For one to three passes, many micro-sized fibers with a few submicron-sized and nano-sized fibers (i.e., CNFs) were seen. After four passes, the sizes of fibers changed from micro-sized and sub-micron-sized to nano-sized fibers due to the shearing force generated by the grinding stone in the disc grinder [1]. Therefore, the morphology of the fibers became narrower and more uniformly nano-sized compared to the results after one and three passes. Figure 10e shows an FE-SEM image containing mostly nano-sized fibers produced after five passes. The results indicate that with additional passes, the sizes of the CNFs became narrower, to about 20 to 50 nm, and the morphology of the fibers became increasingly uniform. 

**Figure 11:**
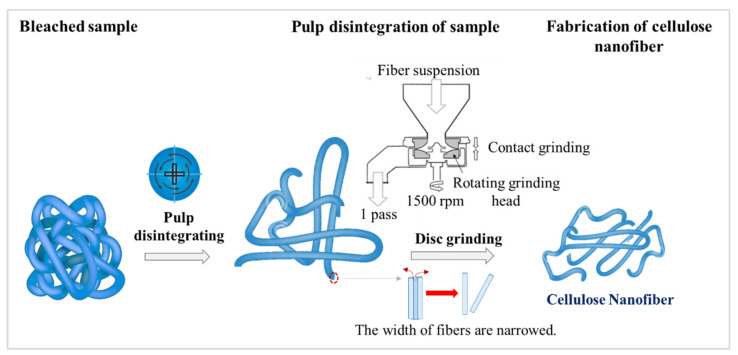
Mechanism of fabricating CNFs using a disc grinder (method A).

The experimental mechanism for the fabrication of the CNFs is shown in Figure 11.

As can be seen in Figure 11, a fibrous suspension was prepared using the pulping process by a pulp disintegrator, and then the CNFs were fabricated by passing the suspension through the disc grinder. In this disc grinding process, the pulp passes through a couple of grinding stones; one grinding stone is fixed and the other stone rotates [23,24]. The fibrillation mechanism of the disc grinder is explained as follows: the cell wall structure, consisting of nanofibers in a multi-layered structure and hydrogen bonds, is broken down by the shearing forces generated by the grinding stones, and then nano-sized fibers are individualized from the pulp fibers. The shearing force generated by the grinding stones degrades the pulp fibers simultaneously with the fibrillation [7,23,24,25]. 

### 3.3. FE-SEM Micrographs of CNFs with Dry Cellulose Powder Using the Disc Grinder

#### 3.3.1. Preparation of Dry Cellulose Powder Using the PBM

This section explains the results of experiments in which a dry cellulose powder was prepared from pretreated cellulose using a PBM. The optimal experimental conditions were determined using a variety of experimental conditions, such as zirconia balls of 3, 5, and 10 mm diameter; a ball to cellulose ratio of 80:1, 120:1, and 160:1; and the rotation speed of 100, 300, and 500 rpm for milling time of 5, 10, and 15 min. The optimal experimental conditions are summarized in Table 1. 

**Figure 12:**
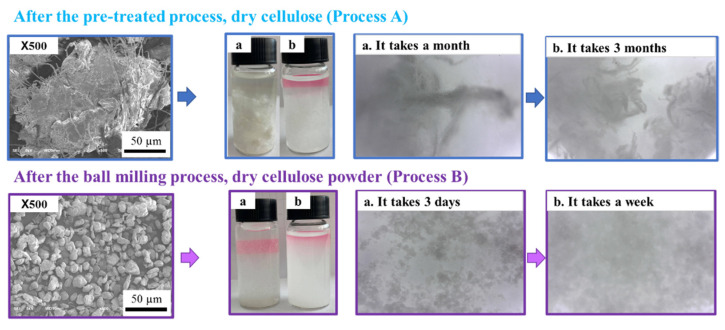
Comparison of cellulose powder and after pretreating the cellulose to form a suspension.

The optimal conditions for the preparation of dry cellulose powder using a PBM were investigated. Under these optimal conditions, a uniformly dry cellulose powder with an average diameter of less than approximately 20 μm was obtained by the PBM (Figure 12). The pulping procedure changed the dry cellulose powder into a suspension, which was then ground into CNFs using a disc grinder.

#### 3.3.2. Comparison Properties of Dry Cellulose Fibers and Dry Cellulose Powder

The next step was the wet disintegration of the pulp cellulose. This disintegration step separates the cellulose fibers of most pulps, including recycled fibers, without changing the structural properties of the cellulose fibers in wet conditions. As a result, a suspension was created to convert the dry cellulose powder to a wet powder. The dry cellulose fibers extracted after the pretreatment process and the dry cellulose powder prepared by PBM were compared in terms of the formation of the suspension and the amount of dissolved water. The dry cellulose fibers and the suspension formation process were examined under a microscope (Dino-Lite, AM3111, Taipei, Taiwan). The results of the formation of the two samples are shown in Table 2. The results showed that the dry cellulose fibers formed a suspension after three months, and a dry cellulose powder formed a suspension after only seven days. 

Figure 12 shows an SEM image and microscopic results of the dry cellulose fibers and dry cellulose powder as they formed suspensions. Figure 12 shows that when the bleached dry cellulose fibers were dissolved in water for one month, no homogeneous suspension was formed, but after three months, a homogeneous suspension formed and the water was absorbed. Figure 12 (method B) shows the results of the dry cellulose powder suspension, and after a week, a homogeneous suspension formed. In this result, the particle size of the dry cellulose decreased, and the morphology changed from fibrous to powder because of fracturing, particle coagulation, and an increased surface area under the optimal experimental conditions during the ball milling process [26]. The reason for the increased surface area is that the amount of chemical activity increased [27]. Therefore, the dry cellulose powder dissolved in water to form a good suspension. As compared to the above results, using dry cellulose powder in the nanofiber production process has the advantages of forming a suspension in a short amount of time and storing cellulose powder with unchanged quality for a long period of time.

#### 3.3.3. Results of FE-SEM Micrographs of CNFs Formed from the Dry Cellulose Powder

**Figure 13:**
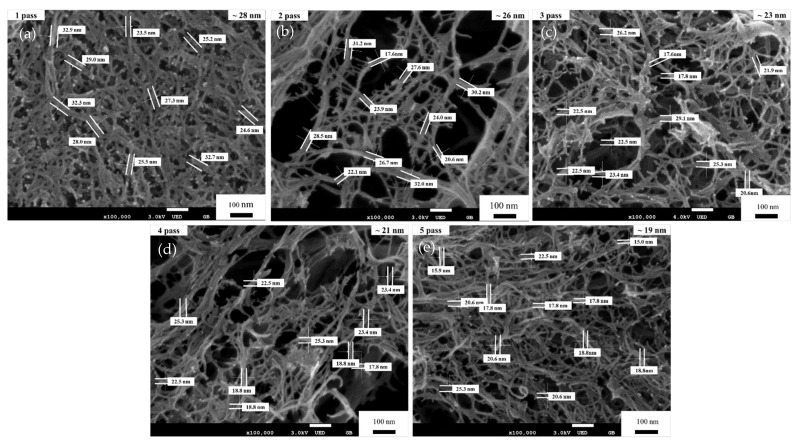
FE-SEM micrographs (×100,000) of CNFs after disc grinding the dry cellulose powder for (**a**) one pass, (**b**) two passes, (**c**) three passes, (**d**) four passes, and (**e**) five passes.

The FE-SEM micrographs in Figure 13 show the microstructures that were obtained when the CNFs were passed one to five times through a disc grinder at 1500 rpm. Most of the CNFs became nano-sized fibers after only one pass (Figure 13a). The CNFs were created by disc grinding dry cellulose powder into uniform nano-sized fibrillated pulp fibers. However, the FE-SEM images after two passes (Figure 13b) show that the CNF’s morphology was uniform, and the width of the fibers had narrowed. Using the disc grinder, the fiber width decreased and narrowed after each pass of the CNF fabrication process. Figure 13e shows FE-SEM micrographs of the pulp fibers fibrillated through the grinder after five passes. The average width of the CNFs was approximately 20 to 30 nm. After five passes, the CNFs were narrower, the fiber structure was more uniform, and the fiber widths were approximately 20 nm, which was the most successful outcome of this study.

**Figure 14:**
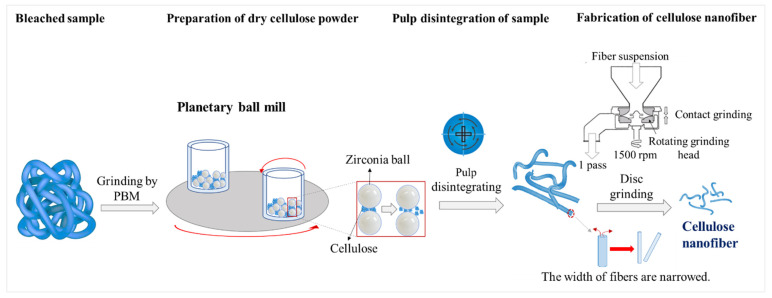
Mechanism of CNF fabrication from dry cellulose powder using a PBM and a disc grinder.

Figure 14 shows the mechanisms of the new method for fabricating CNFs. The mechanisms of the ball milling and disc grinding processes are included in this diagram. The first mechanism is the ball milling grinding mechanism [27]. When the sample is inserted between the balls, two mechanisms are at play: one is impact power, and the other is shear action, where the former affects the volume breakage. The second mechanism is the disc grinding process. Cellulose consists of multi-layered hydrogen bonds that are broken down by the shearing forces generated by the grinding stones, which causes the widths of the fibers to narrow [1,7,23,24,25]. 

Table 3 shows the widths of the CNFs produced using the two different grinding processes (Methods A and B). 

In some research studies [1,28], CNFs with widths of 20–50 nm were fabricated after five passes through a disc grinder. In addition, Iwamoto et al. extracted uniform CNF with a width of 50–100 nm using a disc grinder after 10 passes. Hassan et.al [29] studies showed the fabrication of nanofibers with diameters ranging from 3.5 to 60 nm. The results indicated that the main isolation of nanofibers took place during refining using the ultrafine grinding process, whereas high-pressure homogenization resulted in smaller diameter. As a result of this study, it can be seen that the CNF widths are wider and non-uniform after one pass in method A, and the CNF widths sizes are more uniformly narrow after one pass in method B. For both methods (A and B), the width size of the CNF decreased with an increasing number of passes, and method B was observed to result in more uniform, homogenization of CNF than method A throughout the pass. After five passes, the CNF widths of method A are about 25 nm and those from method B are about 19 nm, and they are more uniform and narrower. The result of this study is that compared with the results of other similar methods of extracting nanocellulose, method B consistently observed relatively good results. Therefore, we discovered a new method for influencing CNFs at the nanoscale: an additional grinding operation utilizing a PBM.

## 4. Conclusions

In this study, we successfully fabricated CNFs from Gelidium amansii using two kinds of grinding processes. As a result of the traditional method (method A), narrow and homogeneous CNFs with widths of 22 nm were fabricated after five passes through a disc grinder. For the new method (method B), we successfully fabricated narrow, uniform, and homogeneous CNFs with a width of 19 nm after five passes through a disc grinder. The preparation of dry cellulose powder using a PBM assisted the CNF fabrication process by limiting changes in the cellulose quality, allowing long-term storage, preparation in a variety of cellulose concentrations, and generation of a good suspension.

## Figures and Tables

**Table 1:** Optimal experimental conditions for the PBM.

Experimental Conditions	Optimal Conditions
Sample	Cellulose
Rotation speed [rpm]	500
Milling time [min]	15
Ball diameter [mm]	3
Ball cellulose ratio [−]	120:1
Ball filling ratio [−]	0.3
Material of media	zirconia

**Table 2:** Comparison of suspension quality.

Type of Sample	Suspension Time			
	3 Days	7 Days	1 Month	3 Months
The dry cellulose fibers	x	x	x	o
The dry cellulose powder	x	o	o	o

x—non-formation of suspension; o—formation of suspension.

**Table 3:** Comparison of the CNF’s width with average size.

Number of Passes	CNF’s Width of Average Size in Process A	CNF’s Width of Average Size in Process B
1 pass	~51 nm	~28 nm
2 passes	~39 nm	~26 nm
3 passes	~34 nm	~23 nm
4 passes	~28 nm	~21 nm
5 passes	~25 nm	~19 nm
